# Diesel Exhaust Emission Soot Coated Pyroelectric Materials for Improved Thermal Energy Harvesting

**DOI:** 10.1002/gch2.201800089

**Published:** 2019-01-02

**Authors:** Puneet Azad, Moolchand Sharma, Rahul Vaish

**Affiliations:** ^1^ Department of Electronics and Communication Engineering Maharaja Surajmal Institute of Technology New Delhi 110058 India; ^2^ School of Engineering Indian Institute of Technology Mandi 175001 Himachal Pradesh India

**Keywords:** diesel exhaust emission, energy harvesting, pyroelectric

## Abstract

Pyroelectric performance is significantly improved using a coating of diesel exhaust soot. Coated pyroelectric sample (lead zirconate titanate) is exposed to a temporal temperature gradient. Under the application of infrared (IR) heating for a given temperature gradient, the maximum open circuit voltage improves more than four times, electric current across a resistance of 10 ohm improves more than six times, and the stored energy in 10 µF capacitor is enhanced by 17 times. These results are important from two aspects: 1) utilization of waste diesel exhaust soot and 2) improving energy harvesting performance of pyroelectric materials.

## 1 Introduction

Waste thermal energy harvesting using pyroelectric materials has gained significant attention of materials research community.^1^, ^2^, ^3^, ^4^, ^5^ This technique seems to have better efficiency than the known other sister techniques such as thermoelectric energy harvesting. Various concepts have been demonstrated for pyroelectric energy harvesting.^6^, ^7^, ^8^ Although results are promising, still these materials are only sufficient for low power electronic devices. Large number of materials such as BaCa*_x_*Ti_1‐_ *_x_*O_3_, Ba_0.85_Ca_0.15_Zr_0.1_Ti_0.9_O_3_, PbZr*_x_*Ti_1‐_ *_x_*O_3_, BaTi_1‐_ *_x_*Sn*_x_*O_3_ have been examined for energy harvesting applications.^2^, ^9^, ^10^, ^11^ In order to improve the performance, several strategies such as inducing porosity, tuning pyroelectric coefficient, and some other physical techniques have been proposed.^1^, ^6^, ^12^, ^13^ Very recently few studies were attempted for surface modification which ultimately improved thermal properties.^14^, ^15^, ^16^ These thermal properties ultimately control heating and cooling of sample as pyroelectric current is directly proportional to heating and cooling rate. A graphene ink–coated polyvinilidenedifluoride (PVDF)‐based pyroelectric harvester enhanced the electrical properties with an increase in pyroelectric current by seven times, open circuit voltage by four times, and harvested energy by four times due to the high thermal radiation absorbance.^17^ Coatings of certain nanocomposite materials such as graphene and carbon on the surface of ferroelectric crystals of lithium niobate (LiNbO_3_) enhanced the performance of pyroelectric materials multifold under the application of solar radiations.^18^ It was shown that the maximum powers obtained were 50 and 90 µW, respectively, for graphene and carbon black coatings over the surface of pyroelectric materials. Further, candle soot–coated lead zirconate titanate (PZT) has shown seven, eight, and 50 times enhancement in open circuit voltage, electric current, and harvested energy, respectively, in comparison to uncoated samples.^19^, ^20^ Abovementioned studies indicated that surface modification of pyroelectric materials could be promising techniques for improving energy harvesting performance. However, some of the abovementioned materials are costly and hence cannot be desirable for application at large scale. In this connection, we decided to explore low‐cost material in view of replacing graphene ink which has been used by Bowen and co‐workers^17^ Diesel exhaust emission is one of undesirable sources of carbon. The hazardous particles present in the diesel exhaust are considered to be dangerous for human health. The inhalation of the exhaust leads to severe diseases such as chronic obstructive pulmonary disease (COPD), asthma, lung cancer, and bronchitis.^21^, ^22^ Several techniques have been introduced over the years to lower down the emissions from the diesel vehicles.^23^, ^24^ In spite of several disadvantages, an attempt has been made to harness the diesel exhaust emission for energy storage electrodes in Li‐ion batteries and supercapacitors by utilizing the carbon and its nanocomposites.^25^ Recently, the blue light present in diesel soot from the internal combustion engines has found photonic applications.^26^ This study comprises utilization of diesel exhaust emission for pyroelectric energy harvesting, which has not been explored till date.

## 2 Results and Discussion

**Figure 1:**
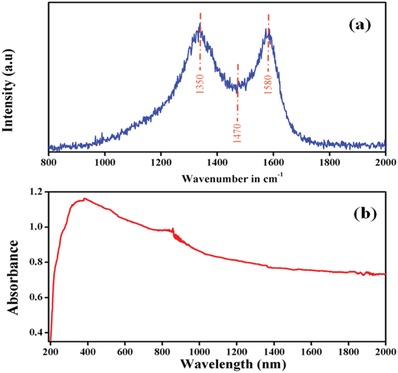
a) Raman spectrum and b) absorbance versus wavelength plot for diesel exhaust emission soot.

**Figure** **1**(a,b) represents the Raman and absorption spectra of diesel exhaust emission soot. Figure 1a represents the Raman spectrum of diesel exhaust emission soot which confirms three typical peaks at 1350, 1470, and 1580 cm^−1^.^27^, ^28^ Vibration modes present different hydrocarbon‐related structure.^29^ Raman band at 1580 cm^−1^ is associated for graphitize amorphous hydrocarbons which come into view as sp^2^ bonding and stretching of C‐C. This peak is calculated by E2g mode of vibration.^30^ Second strong band at 1350 cm^−1^ is assigned for A1g symmetry.^27^ Other week peaks relate to the molecular carbon material of soot.^31^, ^32^ Figure 1b represents absorption of diesel engine exhaust soot from 200 to 2000 nm wavelength. We observed fast absorption of emission from 370 to 1200 nm wavelength range.

**Figure 2:**
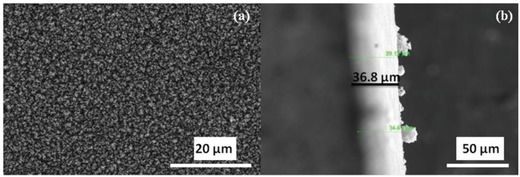
a) Surface morphology and b) SEM image cross‐section along the thickness of diesel exhaust soot ink coated on PZT ceramic buzzer.

**Figure 3:**
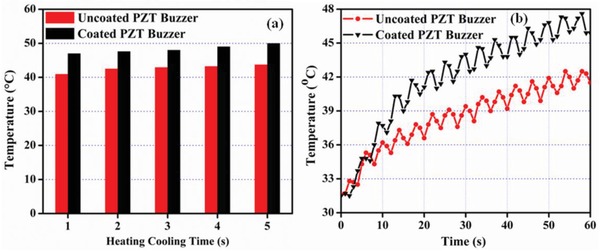
a) Maximum surface temperature for different heating–cooling cycles and b) surface temperature for heating–cooling cycle of 2 s.

**Figure** **2**(a,b) shows surface morphology of diesel exhaust soot ink coated on PZT buzzer. Image shows the uniform coating having submicron particles. Figure 2b shows the cross‐sectional image across the thickness of diesel exhaust soot ink coated PZT sample. Thickness of diesel exhaust soot ink was ≈37 µm on the PZT buzzer surface. There may be enhancements in the electrical parameters with increase in coating thickness. But with further increase in the depth of coatings, the heat may not reach the surface of the pyroelectric material resulting in lower outputs. The uncoated and coated PZT samples were exposed to temporal temperature fluctuations under an infrared (IR) lamp and an arduino switch for time periods from 1 to 5 s. **Figure** **3**a demonstrates the maximum temperature of the uncoated and coated samples for different heating–cooling cycles. It is noted that materials temperature increases with time and reaches at equilibrium after some time as shown in Figure 3b. Mentioned temperature values (in Figure 3a) are equilibrium temperature after 1 min of heating–cooling cycles.

It is illustrated that the maximum and minimum surface temperatures are 50 and 41 °C on uncoated and coated PZT buzzers for heating–cooling cycles of 5 and 1 s. In order to achieve the best performance, there is a need of optimum heating–cooling cycle. It is similar to piezoelectric performance where one can achieve best performance at resonance frequency. Figure 3b shows the surface temperature profile for uncoated/coated sample using heating–cooling cycle of 2 s, which is the optimum cycle in the present case. It is the sample temperature after 60 s of heating–cooling. The temperature goes up and down like ripples due to the alternate heating and cooling. It is clear that the sample coated with diesel exhaust emission attains high temperature as compared to uncoated sample due to high IR absorption.

**Figure 4:**
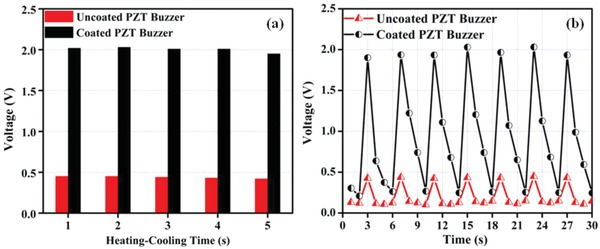
a) Peak open‐circuit voltage for uncoated and coated samples and b) variation of open‐circuit voltage for uncoated and coated samples for a heating–cooling cycle of 2 s.

**Figure** **4**a illustrates the peak open circuit voltage obtained for different heating–cooling cycles with a maximum rise above 2 V in all the cycles. It is shown that the maximum voltages for all the thermal cycles are almost similar with an enhancement of almost four times in voltage across coated samples as compared to uncoated samples. Figure 4b shows the variation of open circuit voltage for a heating–cooling cycle of 2 s. Due to the positive and negative temperature gradient, the voltage rises during heating and falls during cooling, and thereby a periodic waveform is observed across the electrodes. The absorption of heat is less in case of uncoated sample leading to lower voltages across the electrodes of the sample. The peak values of open circuit voltages are found to be 0.45 and 2 V across uncoated and coated samples, respectively, as shown in Figure 4b. Comparing the values, voltage obtained across coated sample is more than four times than that of for uncoated samples.

**Figure 5:**
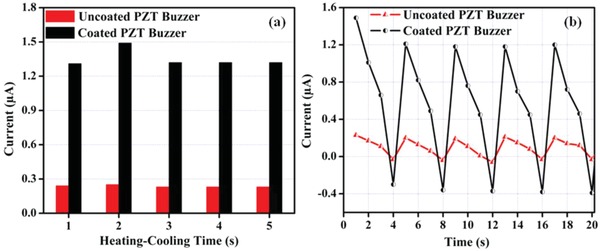
a) Peak pyroelectric current for uncoated and coated samples and b) variation of current for uncoated coated samples for a heating–cooling cycle of 2 s.

**Figure** **5**a shows peak pyroelectric current across 10 Ω resistor for all thermal cycles for all the uncoated and coated samples. The current is observed to be maximum in 2‐2 s heating–cooling cycle and shows an improvement of six times in coated sample as compared to uncoated sample. This could be due to the high IR absorption through black surface of diesel exhaust emission coated on PZT. As shown in Figure 5b, the electric current for heating–cooling cycle of 2 s shows a peak value of 1.49 µA, which stabilizes to 1.2 µA after first cycle. As compared to uncoated sample, which shows peak current of 0.23 µA, the diesel exhaust emission coated sample performs six times better. There may be enhancement in the pyroelectric output with increase in coating thickness. However, after finite thickness, heat will not reach to the surface of the pyroelectric material which will result in lower output.

**Figure 6:**
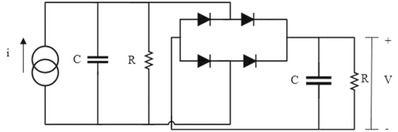
Circuit diagram used in present study.

**Figure 7:**
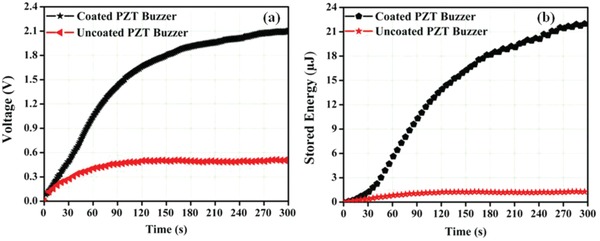
a) Output voltage and b) energy stored in the 10 µF capacitor for uncoated and coated PZT buzzer.

Further, the energy stored in a 10 µF capacitor is demonstrated after conversion of A.C. signals through a bridge rectifier consisting of four Schottky diodes (BAT86) as shown in **Figure** **6** as usually done in several other applications.^33^, ^34^ The output voltage across the capacitor is shown in **Figure** **7**a for uncoated and coated samples over a period of 300 s for heating–cooling cycle of 2 s. The capacitor charges to a maximum voltage of 2.11 V in coated sample in comparison to 0.51 V in uncoated sample. Further, the energy stored across 10 µF capacitor is 17 times enhanced in coated sample (22.26 µJ) as compared to uncoated sample (1.31 µJ). Thus it is shown that diesel exhaust soot increases quantity of heat absorption along with fast dissipation of heat due to large emissivity, which is the ratio of the energy radiated from a material's surface to that radiated from a blackbody. This study shows the potential of diesel exhaust emission–based pyroelectric materials for promising energy harvesting and sensing applications.

## 3 Conclusions

The present study is an experimental investigation for diesel exhaust emission soot coated PZT buzzer for pyroelectric energy harvesting. The electrical outputs across the material are recorded after exposing the surfaces to an IR lamp and flowing water. It is observed that significant enhancements are observed in pyroelectric outputs with the improvements of five times in open circuit voltage, six times in electric current across a 10 Ω resistor, and 17 times in harvested energy. The results suggest the effective use of a waste and harmful automobile exhaust in IR‐sensitive devices and energy harvesting for low‐power electronics.

## 4 Experimental Section

**Figure 8:**
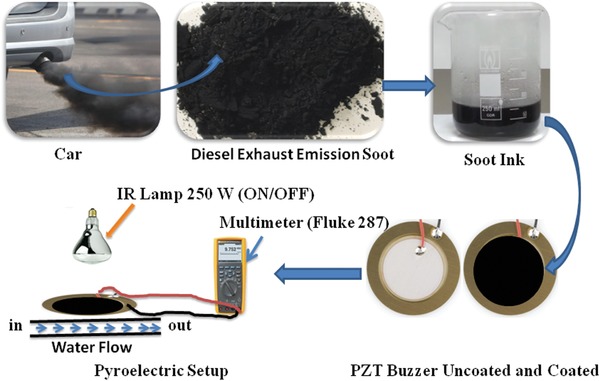
Schematic representations of diesel exhaust emission collection and coating on PZT buzzer along with energy harvesting setup.

The diesel engine exhaust soot ink was fabricated by mixing the exhaust soot (90 wt%) and PVDF (10 wt%) in N‐methyl‐2‐pyrrolidone (NMP). The obtained ink was coated on the electroded surface of PZT buzzer (diameter of upper electrode: 2.5 cm, diameter of metallic part: 3.5 cm) using simple paint brush technique and dried at 50 °C for 2 h in oven. The irradiation area of the sample was ≈5 cm^2^. The typical schematic of the collection and coating of diesel exhaust emission on the PZT buzzer is shown in **Figure** **8**.

The typical thickness of the sample was around 0.2 mm. Similar PZT buzzer was previously used for energy harvesting studies.^35^, ^36^ Two wires were soldered from the upper ceramic portion and lower metallic portion, which acted as electrodes. A Phillips IR lamp (250 W) and a water block were used to generate temperature fluctuations across the upper surface of coated and uncoated samples. Similar setups were close to real time applications and were explored by several researchers.^36^, ^37^, ^38^, ^39^, ^40^, ^41^, ^42^, ^43^ The sample was heated by switching the lamp ON and OFF from 1 to 5 s by placing it 1 cm away from the sample, while it was continuously cooled through water flow (25 °C) below its surface. The variation of surface temperature, open circuit voltage, and current across a 10 Ω resistor were recorded using an electronic true‐rmsmultimeter (Fluke 287). Also, stored energy was recorded using a bridge rectifier, which employed four Schottky diodes (BAT86) and a 10 µF capacitor.

## Conflict of Interest

The authors declare no conflict of interest.
